# Outbreak of *Listeria monocytogenes* in South Africa, 2017–2018: Laboratory Activities and Experiences Associated with Whole-Genome Sequencing Analysis of Isolates

**DOI:** 10.1089/fpd.2018.2586

**Published:** 2019-07-09

**Authors:** Anthony M. Smith, Nomsa P. Tau, Shannon L. Smouse, Mushal Allam, Arshad Ismail, Ntsieni R. Ramalwa, Bolele Disenyeng, Mimmy Ngomane, Juno Thomas

**Affiliations:** ^1^ Centre for Enteric Diseases, National Institute for Communicable Diseases, Division of the National Health Laboratory Service, Johannesburg, South Africa.; ^2^ Department of Clinical Microbiology and Infectious Diseases, Faculty of Health Sciences, University of the Witwatersrand, Johannesburg, South Africa.; ^3^ Sequencing Core Facility, National Institute for Communicable Diseases, Division of the National Health Laboratory Service, Johannesburg, South Africa.

**Keywords:** Listeria monocytogenes, whole-genome sequencing, sequence type 6, ST6, outbreak, listeriosis, South Africa

## Abstract

In South Africa, a progressive increase in listeriosis cases was noted from mid-June 2017, heralding what was to become the world's largest listeriosis outbreak. A total of 1060 cases were reported for the period January 1, 2017 to July 17, 2018. We describe laboratory activities, experiences, and results of whole-genome sequencing (WGS) analysis of *Listeria monocytogene*s isolates associated with this outbreak. Bacteria were identified using the VITEK-2 COMPACT 15 microbial identification system. WGS was performed using Illumina MiSeq technology. WGS data were analyzed using CLC Genomics Workbench Software and free-to-use on-line analysis tools/pipelines. Multilocus sequence typing (MLST) showed that 91% of clinical isolates were sequence type 6 (ST6), determining that the outbreak was largely associated with *L. monocytogene*s ST6. Epidemiological and laboratory findings led to investigation of a large ready-to-eat processed meat production facility in South Africa, named Enterprise Foods. *L. monocytogenes* ST6 was found in environmental sampling swabs of the production facility and in ready-to-eat processed meat products (including polony, a product similar to bologna sausage) manufactured at the facility. ST6 isolates, sourced at the Enterprise Foods production facility and from Enterprise food products, were shown by single nucleotide polymorphism (SNP) analysis to be highly related to clinical isolates; these nonclinical ST6 isolates showed <10 SNP differences when compared to clinical ST6 isolates. Core-genome MLST showed that clinical ST6 isolates and Enterprise-related ST6 isolates had no more than 4 allele differences between each other, suggestive of a high probability of epidemiological relatedness. WGS data interpreted together with epidemiological data concluded that the source of the listeriosis outbreak was ready-to-eat processed meat products manufactured by Enterprise Foods. Listeriosis has now been added to the South African list of mandatory notifiable medical conditions. Surveillance systems have been strengthened to facilitate prevention and early detection of listeriosis outbreaks.

## Introduction

*L**isteria monocytogenes* is a Gram-positive bacterium that causes the disease named listeriosis. Acquisition of the *L. monocytogenes* pathogen occurs mainly by consumption of contaminated food. Infections with *L. monocytogenes* can result in mild febrile gastroenteritis in healthy individuals; however, invasive disease characterized by bacteremia, meningitis, pneumonia, endocarditis, and sepsis can occur, particularly in high risk groups (Ferreira *et al.*, 2014). The pathogen most commonly affects immuno-compromised individuals, pregnant women, neonates, and the elderly. Listeriosis is associated with case fatality rates as high as 30% (Lomonaco *et al.*, 2015).

Recently, there have been several international reports of *L. monocytogenes* associated foodborne disease outbreaks. These outbreaks have been linked to a variety of different food vehicles, including ice cream (Chen *et al.*, 2017b; Li *et al.*, 2017), ready-to-eat meat products and meat pâté (Kvistholm Jensen *et al.*, 2016; Althaus *et al.*, 2017; Gelbicova *et al.*, 2018), ready-to-eat fish (Nakari *et al.*, 2014; Schjorring *et al.*, 2017), stone-fruits (Chen *et al.*, 2016), cheese (Chen *et al.*, 2017a), and caramel-apples (Angelo *et al.*, 2017). Multilocus sequence typing (MLST) of isolates associated with outbreaks has shown that several sequence types (STs) of *L. monocytogenes* are capable of causing outbreaks. *L. monocytogenes* sequence type 6 (ST6) is one of the *L. monocytogenes* subtypes that have been associated with outbreaks. In 2013, a U.S. multistate cheese outbreak was associated with *L. monocytogenes* ST6 (www.cdc.gov/listeria/outbreaks/cheese-07-13). In 2016, meat pâté contaminated with *L. monocytogenes* ST6 caused an outbreak in Switzerland (Althaus *et al.*, 2017). More recently, in early 2018, reports emerged of a *L. monocytogenes* ST6 outbreak (ongoing since 2015) in five European Union member states (Austria, Denmark, Finland, Sweden, and the United Kingdom); the outbreak was linked to frozen corn and possibly to other frozen vegetables (https://ecdc.europa.eu/en/news-events/listeria-monocytogenes-outbreak-47-cases-including-9-deaths).

In South Africa, comprehensive historic data for *L. monocytogenes* are lacking. This includes a lack of data related to prevalence, epidemiology, and description of clusters/outbreaks. The first documented report of a human listeriosis outbreak in South Africa, occurred over the period of August 1977 to April 1978; the outbreak occurred in the Gauteng Province of South Africa and involved 14 cases (Jacobs *et al.*, 1978). In 1999, in the Mpumalanga Province of South Africa, an outbreak of listeriosis was described in cattle and sheep that were fed poor quality unmarketable potatoes (Van Vollenhoven, 1999). More recently, some reports from South Africa have described the presence of *L. monocytogenes* in a variety of human food items (Nel *et al.*, 2004; van Nierop *et al.*, 2005; Plessis *et al.*, 2017). Notably, in 2015, a cluster of human cases caused by *L. monocytogenes* ST6 was described in the Western Cape Province of South Africa (Smith *et al.*, 2016).

**FIG. 1.**
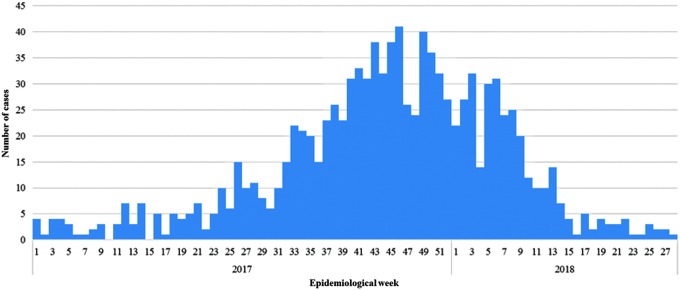
Epidemiological curve of laboratory-confirmed listeriosis showing number of cases by week (listed according to date of sample collection), South Africa, January 1, 2017 to July 17, 2018 (*n* = 1060).

From mid-June 2017, a marked, progressive increase in listeriosis cases was noted in South Africa, which heralded the onset of an outbreak. A staggering total of 1060 cases were reported for the period January 1, 2017 to July 17, 2018 (Fig. 1). The World Health Organization (WHO) described this as the largest listeriosis outbreak that had ever been detected worldwide. The molecular epidemiology of the outbreak was investigated using whole-genome sequencing (WGS) analysis of the *L. monocytogene*s isolates; WGS was performed in as real time as possible. This was a massive undertaking for a relatively small reference public health laboratory in South Africa, the Centre for Enteric Diseases (CED), a laboratory that was still in its WGS “infancy” and still in the process of taking “small steps” toward the implementation of WGS for analysis of enteric pathogens. Herewith, we describe the CED laboratory activities, experiences and results of WGS analysis of *L. monocytogene*s isolates associated with the largest ever reported outbreak of listeriosis globally.

## Materials and Methods

### Outbreak detection and public health response

During mid-June 2017, the National Institute for Communicable Diseases (NICD) was notified of an unusual increase in listeriosis cases at public sector hospitals in one province of South Africa. The NICD's CED and Outbreak Response Unit, launched an investigation into the reported increase in cases. A listeriosis outbreak was later declared. The CED focuses on surveillance and public health-focused research of pathogens associated with diarrhea and enteric fevers, and actively assists with the investigation and response to enteric disease outbreaks (including foodborne and waterborne disease outbreaks). The center provides specialized reference laboratory testing for enteric bacteria and viruses, including potential causes of outbreaks. It is a source of strategic data, technical support, and policy advice to the South African Department of Health and other major stakeholders, and contributes its expertise toward strengthening outbreak preparedness and response to public health emergencies.

### Referral of isolates to the CED

Preceding this listeriosis outbreak, clinical microbiology laboratories across South Africa would forward *L. monocytogenes* isolates to the CED on an *ad hoc* basis. Upon declaration of the outbreak, all diagnostic microbiology laboratories across the country were formally requested to forward all isolates to the CED for further characterization. These included microbiology laboratories in all fields and disciplines, laboratories performing testing on every type of specimen, such as those sourced from clinical (humans), animals, food, food production facilities, environment, and others. The CED then performed phenotypic and genotypic characterization of all isolates.

### Phenotypic identification of bacteria

Bacteria were received on Dorset-Egg transport media (Diagnostic Media Products [DMP]; National Health Laboratory Service, Johannesburg, South Africa) and sub-cultured onto 5% Blood Agar (DMP), to check viability and purity. The identification of bacterial cultures was confirmed using the VITEK-2 COMPACT 15 automated microbial identification system (bioMérieux, Marcy-l'Étoile, France).

### Genomic DNA isolation from bacteria and WGS

Genomic DNA was isolated from bacteria using a Qiagen QIAamp DNA Mini Kit (QIAGEN, Hilden, Germany). DNA was sequenced using Illumina MiSeq (Illumina, San Diego, CA) next generation sequencing technology; DNA libraries were prepared using a Nextera XT DNA Library Preparation Kit (Illumina), followed by 2 × 300 paired-end sequencing runs with ∼70 times coverage. Raw sequencing data (FastQ files for paired-end reads) were analyzed using tools available in the CLC Genomics Workbench Software, version 11 (CLC bio, Aarhus, Denmark); using the “trim sequences tool,” sequence reads were trimmed to include quality trimming and ambiguity trimming, and length trimming to discard reads below a length of 100 bases; trimmed reads were assembled using the “*de novo* assembly tool.”

### MLST of bacteria

Assembled genome data were analyzed using the “MLST” on-line analysis pipeline available at the Center for Genomic Epidemiology (CGE) of the Technical University of Denmark (Larsen *et al.*, 2012). MLST produces STs based on sequence analysis of seven housekeeping genes, as described at the Bacterial Isolate Genome Sequence Database (BIGSdb) for *L. monocytogenes* (BIGSdb-*lm*) database (http://bigsdb.pasteur.fr/listeria/listeria.html).

### Single nucleotide polymorphism profiles and phylogenetic analysis of bacteria

Assembled genome data were analyzed using the “CSIPhylogeny 1.4” on-line analysis pipeline available at the CGE (Kaas *et al.*, 2014). The CSIPhylogeny pipeline uses various publicly available programs; the analysis steps are briefly described as follows: assembled genome data are aligned against a reference genome and single nucleotide polymorphisms (SNPs) are called; SNPs are filtered and qualified; final qualified SNPs for each genome is concatenated to an alignment; phylogeny is then inferred based on a comparison of SNP alignments of strains. SNPs were called by alignment and referencing against a South African *L. monocytogenes* ST6 isolate (reference No. YA00053238). SNP alignments were analyzed with iTOL software (http://.itol.embl.de) to generate phylogenetic maximum-likelihood trees.

### Core-genome multilocus sequence typing and phylogenetic analysis of bacteria

Raw sequencing data (FastQ files for paired-end reads) were further analyzed at the BIGSdb-*lm* database, to confirm MLST profiles of isolates and to determine the core-genome multilocus sequence typing (cgMLST) profiles of isolates. The cgMLST scheme is based on 1748 genes (Moura *et al.*, 2016). Data (including cgMLST allele data) were exported from the BIGSdb-*lm* and then further captured into a BioNumerics Software version 7.6 database (Applied Maths, Sint-Martens-Latem, Belgium). Within this BioNumerics platform, phylogenetic cluster analysis of isolates was investigated using cgMLST data (categorical data values) analyzed using a single linkage algorithm; cluster analysis was depicted using a minimum spanning tree.

### *In silico* analysis of sequencing data

Tools built into the BIGSdb-*lm* were used for further *in silico* analysis of sequencing data. These included tools for polymerase chain reaction (PCR) serogrouping.

### Data availability

Genome sequences for 10 *L. monocytogenes* ST6 isolates associated with this South African outbreak have been deposited at National Center for Biotechnology Information (NCBI)/GenBank under the accession numbers QEXB00000000 to QEXK00000000 (BioProject No. PRJNA451422, BioSample Nos. SAMN08970424 to SAMN08970415, respectively) (Allam *et al.*, 2018).

## Results and Discussion

### CED, PulseNet Africa and readiness for WGS analysis of *L. monocytogenes*

The CED is a member of the regional PulseNet Africa laboratory network, which forms part of the PulseNet International network (www.pulsenetinternational.org), a global molecular subtyping network for foodborne disease surveillance. In addition, the CED also coordinates the PulseNet Africa Network. PulseNet Africa was established in August 2010. Currently, formal membership includes 12 countries (South Africa, Kenya, Senegal, Cameroon, The Gambia, Malawi, Tanzania, Cote d'Ivoire, Ghana, Uganda, Mozambique, and Nigeria). Communication and discussion in the Network is achieved via a ListServ communication platform. The CED runs training courses in molecular subtyping methodologies, including pulsed-field gel electrophoresis (PFGE) analysis and multiple-locus variable-number tandem-repeats analysis (MLVA). The CED offers continuous assistance, advice, and guidance to African countries with regards to molecular subtyping of enteric pathogens; this includes assistance with PFGE analysis of isolates. The CED hosts/manages an “African” molecular subtyping database that includes hundreds of PFGE patterns from enteric pathogens isolated across Africa.

The CED has published extensively on the use of molecular subtyping methodologies for routine surveillance activities and for investigation of outbreaks involving enteric pathogens. This has included the use of PFGE analysis (Smith *et al.*, 2011; Tau *et al.*, 2012; Ismail *et al.*, 2013), MLVA (Muvhali *et al.*, 2017; Tau *et al.*, 2017) and MLST (Smith *et al.*, 2014, 2017). In late 2015, we were ready to take the first step toward the use of WGS for analysis of enteric pathogens. Our institution (the NICD) had just established a Sequencing Core Facility equipped with Illumina MiSeq next-generation sequencing equipment. Armed with CLC Genomics Workbench Software and various free-to-use on-line WGS analysis tools/pipelines, we were ready to commence with analysis of WGS data. In September 2015, we initiated our WGS activities by investigating a cluster of listeriosis cases reported from the Western Cape Province of South Africa (Smith *et al.*, 2016). This analysis was timely, as the steering committee of the PulseNet International network was in discussions to start with implementation of WGS, of which the networks vision for implementation of WGS was recently published in 2017 (Nadon *et al.*, 2017).

### Listeriosis outbreak, description of clinical *L. monocytogenes* isolates and the ST6 outbreak strain

During mid-June 2017, several clinicians and microbiologists reported unusually high numbers of listeriosis cases at a number of sites in Gauteng Province of South Africa. A listeriosis outbreak was confirmed, with a total of 1060 cases eventually reported for the period January 1, 2017 to July 17, 2018 (Fig. 1). For the years 2013 to 2016, a range of 55–113 laboratory-confirmed cases of listeriosis occurred annually in South Africa. At the peak of the outbreak (mid-November 2017), 41 listeriosis cases were reported in a single week. Cases were reported from across the country; however, most cases were reported from Gauteng Province (58%), followed by Western Cape (13%) and KwaZulu-Natal (8%) provinces. Women accounted for 55% of the patients. The ages of patients ranged from birth to 93 years (median 19 years). Neonates (aged ≤28 d) were the most affected age group, accounting for 43% of cases. This was followed by adults of 15–49 years of age, accounting for 32% of cases. Final outcome was known for 806/1060 (76%) of cases; 27% (216/806) with known outcome died. These outcome data are in agreement with previously reported listeriosis case-fatality rates that can reach 30% (Lomonaco *et al.*, 2015).

Laboratory analysis identified the causative species as *L. monocytogenes*. At the onset of the outbreak, we decided to investigate all bacterial isolates using WGS, with WGS data analysis conducted using various free-to-use on-line WGS analysis tools/pipelines. We certainly did not anticipate the mammoth task that awaited us. The weekly number of *L. monocytogenes* isolates received at our laboratory multiplied rapidly, increasing 40-fold at the height of the outbreak. The laboratory staff (two technicians, two technologists, and two scientists) were rapidly overwhelmed with the increased workload. WGS was performed in as real time as possible. From isolate reception at CED to WGS data availability, the timeline ranged from 7 to 10 working days. WGS was performed on 636 clinical isolates recovered over the period January 1, 2017 to July 17, 2018. MLST of WGS data showed that 91% (576/636) of isolates belonged to MLST ST6, while the remainder belonged to 14 different STs (ST1, ST101, ST155, ST2, ST204, ST219, ST224, ST3, ST5, ST54, ST7, ST8, ST87, and ST876). This determined that an ST6 strain was the most likely cause of the outbreak. The ST6 isolates were also found to belong to serogroup IVb, as determined by *in silico* PCR serogrouping. *L. monocytogenes* ST6 has previously been reported to be associated with outbreaks (Althaus *et al.*, 2017). This ST6 subtype has also been described to be associated with an increased rate of unfavorable outcomes in patients with meningitis (Koopmans *et al.*, 2013).

### Source of the outbreak and analysis of non-human *L. monocytogenes* isolates

By early January 2018, food history interviews with patients suggested that “polony” was among the most commonly consumed foodstuff among persons with listeriosis. Polony is a ready-to-eat processed meat product, similar to bologna sausage. Epidemiological and laboratory findings led to the investigation of a large ready-to-eat processed meat production facility in South Africa, named Enterprise Foods. On February 2, 2018, the production facility was inspected and numerous environmental sampling swabs were collected throughout the facility. *L. monocytogenes* ST6 was isolated from the environment of numerous areas of the production facility, including postcooking areas. The same ST6 strain was also found in several food products (including polony) manufactured at the facility. On March 4, 2018, a recall of affected food products was initiated and Enterprise Foods' production facilities were shut down.

### Phylogenetic analysis of *L. monocytogenes* isolates using SNP analysis and cgMLST

**FIG. 2.**
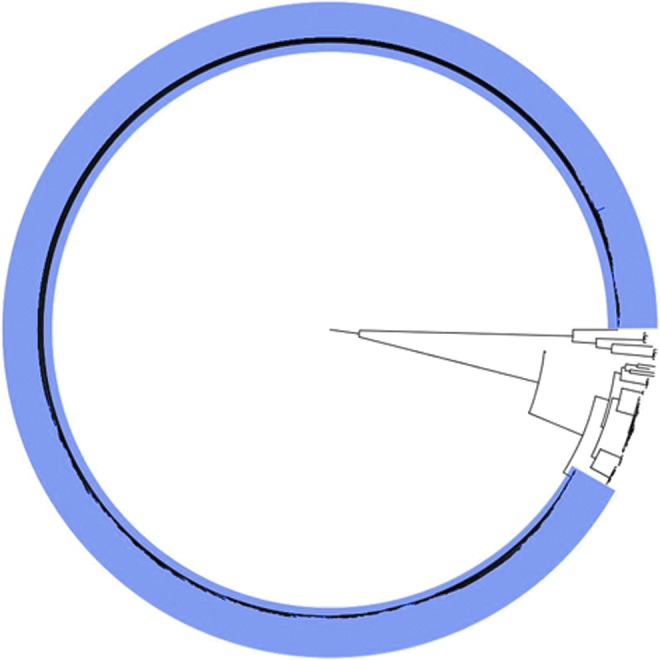
Maximum likelihood tree (circular tree) drawn using SNP alignments from WGS data of clinical *Listeria monocytogenes* isolates. The ST6 outbreak cluster representing 91% of the clinical isolates is highlighted with shading. SNP, single nucleotide polymorphism; ST6, sequence type 6; WGS, whole-genome sequencing.

SNP analysis of clinical isolates showed that the ST6 isolates all clustered together on a phylogenetic tree (Fig. 2). Furthermore, almost all (99%) of the ST6 isolates were all highly related, with SNP differences ranging from zero to nine. ST6 isolates, sourced at the Enterprise Foods Polokwane production facility and from Enterprise Foods products, were shown by SNP analysis to be highly related to the clinical isolates; these nonclinical ST6 isolates showed <10 SNP differences when compared to clinical ST6 isolates. Similar SNP differences and clonal characteristics have previously been reported in studies describing *L. monocytogenes* foodborne outbreaks (Orsi *et al.*, 2008; Kvistholm Jensen *et al.*, 2016).

**FIG. 3.**
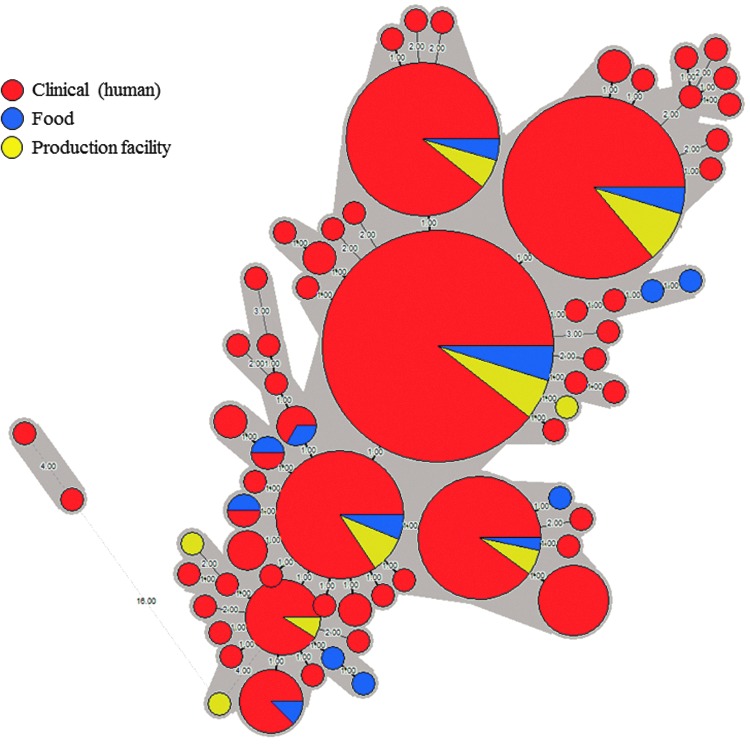
Minimum spanning tree drawn using cgMLST data from 374 *Listeria monocytogenes* ST6 isolates from several sources including clinical (human), food, and production facility. The circular nodes represent isolate(s) having the identical cgMLST profile; the larger the node, the more isolates are reflected. The number values between adjacent nodes indicate the number of allele differences between nodes. Gray shading encompasses all nodes (isolates) that have no more than four allele differences when compared to their adjacent neighboring nodes (isolates). The source of isolates is reflected in the coloring of the nodes. cgMLST, core-genome multilocus sequence typing.

For cgMLST analysis of ST6 isolates from all sources, raw sequencing data were submitted to the BIGSdb-*lm* hosted by Institut Pasteur. BIGSdb-*lm* only accepts sequencing data that passes their strict quality control checks. To date, for ST6 isolates recovered over the years 2017–2018, sequencing data for 374 isolates have passed BIGSdb-*lm* quality control checks; these data were further investigated using cgMLST. With the exception of two clinical ST6 isolates recovered in 2017, all the remaining ST6 isolates (*n* = 372), had no more than four allele differences between each other, when investigated using cgMLST analysis (Fig. 3). This indicated that these 372 ST6 isolates were highly genetically related and suggested a high probability of epidemiological relatedness of the ST6 isolates, as concluded following analysis using this cgMLST scheme described by Moura *et al.* (2016, 2017). The BIGSdb-*lm* assigned these 372 ST6 isolates a cgMLST profile number 4148 (CT4148), a profile unique to our South African isolates and not reported from any other country worldwide. These 372 ST6 (CT4148) isolates included 326 clinical isolates, 22 Enterprise food isolates, and 24 environmental isolates recovered from Enterprise Foods Polokwane production facility (Fig. 3).

## Limitations of the Data Described in This Article

The data described in this article are preliminary and so has limitations. The data described are a prelude to more detailed articles that will soon follow. All epidemiological aspects of this outbreak investigation have not been presented and discussed—these aspects will be the subject of a larger “epidemiology article” that will soon follow and be published separately. Likewise, all molecular and genomic aspects of the *L. monocytogenes* isolates have not been presented and discussed—these aspects will be the subject of a larger “molecular article” that will soon follow and be published separately.

## Conclusions

WGS data interpreted together with epidemiological data have determined that the source of the listeriosis outbreak in South Africa was ready-to-eat processed meat products manufactured by Enterprise Foods. This was the largest ever reported outbreak of listeriosis globally. Outbreak milestones included an alert in mid-June 2017, a peak in mid-November 2017, and finally the identification of the outbreak source in mid-February 2018. This 8-month timeline was rather remarkable, considering the large number of cases involved and the limited capacity and resources available for foodborne disease outbreak investigations in South Africa. As a direct result of this outbreak, listeriosis has now been added to the South African list of mandatory notifiable medical conditions. Surveillance systems have been strengthened to facilitate prevention and early detection of listeriosis outbreaks.
